# Effects of Combined Application of Different Nitrogen Forms on Substrate Nutrient Utilization, Root Microenvironment, and Tomato Yield

**DOI:** 10.3390/microorganisms14010158

**Published:** 2026-01-10

**Authors:** Shuyan Jiang, Jianhong Sun, Ning Jin, Shuya Wang, Shuchao Huang, Zhaozhuang Li, Jihua Yu, Jian Lyu, Li Jin

**Affiliations:** 1 College of Horticulture, Gansu Agricultural University, Lanzhou 730070, China; 2 State Key Laboratory of Aridland Crop Science, Gansu Agricultural University, Lanzhou 730070, China

**Keywords:** forms of nitrogen, tomato yield, plant mineral elements, substrate nutrients, substrate microorganisms

## Abstract

In facility tomato production, the excessive application ratio of ammonium nitrogen (NH_4_^+^-N) often leads to root acidification and calcium-magnesium antagonism. Although amide nitrogen (urea-N) has better buffering properties, it needs to be hydrolyzed before utilization, resulting in a lag effect. Previous studies have mostly focused on a single nitrogen source or a fixed proportion, and there is still a lack of systematic evidence on the nitrogen supply effects of different nitrogen application combinations at different growth stages of tomatoes. Therefore, in this experiment, tomato cultivar ‘Jingfan 502’ was used. All treatments received the same total nitrogen concentration (15 mM), but the nitrogen was supplied as different combinations of ammonium nitrogen (AN) and amide nitrogen (UN). Six AN–UN ratio treatments were designed: CK (0% AN, 0% UN), T1 (100% AN, 0% UN), T2 (0% AN, 100% UN), T3 (25% AN, 75% UN), T4 (50% AN, 50% UN), and T5 (75% AN, 25% UN). T3 (25% NH_4_^+^ + 75% urea) increased single-plant yield by 64.04% and 5.10%, and total N, P, K, and Mg accumulation by 29.0% and 20.7%, relative to T1 and T2. In addition, compared to T1 and T2, the nitrogen fertilizer uptake rate of the T3 treatment increased by 17.00% and 24.90%, respectively, and the electrical conductivity (EC) increased by 27.04% and 44.84%, respectively. Redundancy Analysis (RDA) showed that enzyme activities, total N and electrical conductivity were positively linked to microbial communities in T3 and T4, whereas communities in CK, T1, T2 and T5 correlated with nutrients and pH. Under controlled pot conditions, T3 optimizes the rhizosphere micro-environment, enhances microbial abundance and nutrient uptake, and provides a theoretical basis for precise N management in tomato.

## 1. Introduction

Tomato (*Solanum lycopersicum* L.) is an annual or perennial herbaceous plant belonging to the genus *Lycopersicum* in the family Solanaceae, native to South America. It is nutritionally rich, containing carbohydrates, vitamin C, lycopene, and other health-beneficial substances, which positively impact human health and are thus highly favored by consumers [1]. Tomato is the third most important vegetable crop worldwide, with an annual planting area of 4.73–5.00 million hectares and a global output of 186 million t (fresh weight), averaging 3.7–3.8 kg m^−2^. Based on international wholesale prices in 2022, the world tomato industry generates more than USD 120 billion per year, with China being the largest producer [2].

However, the high quality and yield of tomatoes rely on scientific planting management, among which the rational regulation of nitrogen plays a decisive role [3]. Nitrogen is a core element for plant growth and development, and its forms directly affect yield and quality [4]. In agricultural production, ammonium nitrogen (NH_4_^+^), nitrate nitrogen (NO_3_^−^), and urea-N (urea) are the main nitrogen sources. Studies have shown that mixed nitrogen sources can significantly increase crop yield through synergistic effects, but their ratios still need further exploration [5]. Excessive ammonium nitrogen can lead to soil acidification and root toxicity in plants [6]. Excessive nitrate nitrogen can cause substantial gas emissions and water pollution due to nitrogen leaching [7]. Excessive urea-N can result in soil compaction and reduced fertility [8], so adopting rational fertilization measures is of great significance. The combined application of different nitrogen forms can produce different physiological effects on plant growth [9,10]. For example, when NH_4_^+^ and NO_3_^−^ are applied in combination, tea plants often grow better and have higher biomass [9]. Chen et al. [11] found that a 1:1 ratio of ammonium nitrogen to nitrate nitrogen can optimize the growth and some quality indices of tobacco. In tomato, raising the NH_4_^+^ proportion in the nutrient solution from 0% to 50% markedly shortened primary-root length, decreased lateral-root number and reduced root surface area, with 10 mmol L^−1^ NH_4_^+^ almost completely suppressing lateral-root formation. By contrast, a NO_3_^−^/NH_4_^+^ ratio of 75:25 produced the highest photosynthetic rate, largest leaf area and greatest single-plant yield, outperforming the ammonium-only treatment by more than 18% [12]. The ratios of nitrogen forms in the soil not only directly affect plant metabolism but also indirectly regulate plant nutrient uptake by altering soil physicochemical properties and nutrient cycling efficiency [13]. For example, the nitrification of NH_4_^+^ can significantly influence soil pH and redox potential, thereby changing the bioavailability of mineral elements such as phosphorus and potassium, while the accumulation of NO_3_^−^ can promote root absorption of cations like calcium and magnesium through osmoregulation [14]. Moreover, the transformation processes of different nitrogen forms are closely related to soil carbon and nitrogen metabolism. The assimilation of NH_4_^+^ consumes a large amount of carbon skeletons, which may accelerate the decomposition of soil organic matter, while the reduction of NO_3_^−^ consumes photosynthetic assimilates, affecting the allocation of carbon to roots [15]. Studies have also found that nitrogen fertilization can affect soil microbial community structure and soil enzyme activity [16]. Soil microorganisms are important components of the soil ecosystem, where bacteria and fungi, which account for a large proportion of soil microorganisms, determine most of the functions of soil microorganisms [17]. Enzymes in soil are involved in soil nutrient cycling and plant nutrient supply [18], and the higher their activity, the more they can promote plant growth and development [19]. Therefore, soil enzyme activity and soil microbial counts can serve as important indicators of soil microecological health [20]. For example, compared to NO_3_^−^, NH_4_^+^ is more easily absorbed and utilized by plants and microorganisms to promote biomass increase [21]. The combined application of ammonium nitrogen and nitrate nitrogen can increase soil organic matter and accelerate nutrient cycling by microorganisms, thereby promoting crop growth [22].

As mentioned above, most studies on different nitrogen forms have focused on the ratios of nitrate nitrogen to ammonium nitrogen. Currently, there are few studies on the combined application of ammonium and ureide nitrogen at different ratios to ensure adequate nitrogen supply throughout the entire growth period of tomatoes. Therefore, this experiment investigated the effects of different ratios of ammonium nitrogen and urea-N on tomato yield, mineral elements, substrate microbial communities, and substrate enzyme activities, with the aim of providing the optimal nitrogen fertilizer structure and ratio for tomato growth and improving substrate nutrient conditions. Our hypotheses are as follows: (i) Compared to a single nitrogen source, the combined application of ammonium and amide nitrogen will increase tomato yield and nutrient accumulation. (ii) Compared to a single nitrogen source, the combined application of ammonium and amide nitrogen will alter substrate enzyme activity and the microbial community.

## 2. Materials and Methods

### 2.1. Plant Materials and Growth Conditions

The experiment was conducted in a glass greenhouse located at the College of Horticulture, Gansu Agricultural University, Lanzhou, China (36°05′39.86″ N, 103°42′31.09″ E), using ‘Jinfan 502’ tomato as the experimental plant material. The tomato seeds (‘Jinfan 502’) are provided by the Gansu Academy of Agricultural Sciences, Lanzhou, China. First, soak the seeds in a 10% sodium hypochlorite solution for 5 min, and then in hot water at 50–55 °C for 25–30 min for disinfection. Soak at room temperature for 8 h. After soaking, transfer the seeds to a 28 °C incubator for germination. When the germination rate reached 80%, the seedlings were transplanted into 50-cell trays filled with 10 g of seedling substrate per cell. After 30 days of seedling culture (4–5 true leaves), the plants were transplanted into cultivation pots (20 cm × 30 cm) for management. Fill each basin with 2.5 kg (±50 g) of air-dried substrate. Plant two seedlings of similar size in each pot. The environmental conditions for plant growth were as follows: temperature of 30 ± 2 °C/20 ± 2 °C (day/night), photoperiod of 12 h/12 h (day/night), and relative humidity of 60% to 70%. The physicochemical properties of the substrate are shown in Table 1.

### 2.2. Experimental Design

Our experimental design follows a previous study reported by our group [23], which set up 12 treatments (T1–T12) with three nitrogen forms applied in combination and a control (CK) without nitrogen fertilizer. The nutrient solution used in the experiment was prepared based on Hoagland nutrient solution, with the nitrogen forms in the Hoagland nutrient solution formula adjusted, while the contents of other elements remained unchanged. This experiment reduced the number of treatments by 7, focusing on the ratio of ammonium nitrogen to amide nitrogen. The nitrogen forms in the Hoagland nutrient solution formula were adjusted to ammonium nitrogen and amide nitrogen, while the contents of other elements remained unchanged. One week after planting, the plants were irrigated with nutrient solution every three days, with 1000 mL of nutrient solution each time. In this experiment, the nitrogen-free treatment (CK) was used as the control, and different nitrogen form ratios (T1–T5) were set under the same conditions of N (15 mM), P (1 mM), K (6 mM), Mg (2 mM) and Ca (5 mM). Ammonium nitrogen was supplied by (NH_4_)_2_SO_4_, purchased from Tianjin Zhiyuan Chemical Reagent Co., Ltd. (Tianjin, China), and urea-N was supplied by urea, purchased from Anyang Zhongying Chemical Fertilizer Co., Ltd. (Anyang, China). MgSO_4_·7H_2_O was used as the magnesium source, KH_2_PO_4_ as the phosphorus source, KCl and KH_2_PO_4_ as the potassium sources, CaCl_2_ as the calcium source, and the micronutrients were supplied according to the Hoagland nutrient solution. To prevent the transformation of ammonium ions into nitrate ions in the nutrient solution, a nitrification inhibitor, dicyandiamide (C_2_H_4_N_4_, 7 μmol L^−1^), was added to the nutrient solution. According to the nutrient absorption characteristics of tomatoes throughout their growth period, the ratio of their requirements for N, P, K, Ca and Mg is approximately 1:0.29:1.6:1.2:0.26 [24]. Given a total nitrogen application rate of 300 kg hm^−2^ and a planting density of 37,500 plants per hectare, the fertilizer application rate per plant was calculated. On average, each plant received approximately 8.00 g of pure N, 2.32 g of P, 12.80 g of K, 9.60 g of Ca, and 2.08 g of Mg. Ensure that the nutrient supply for each treatment is sufficient and consistent, thereby eliminating the interference of non-nitrogen factors on the test results. The experimental treatments are shown in Table 2.

### 2.3. Measured Indices and Methods

#### 2.3.1. Tomato Yield Determination

Yield per plant: Five tomato plants were selected and marked for each treatment. After the fourth fruit cluster set, the terminal bud of the tomato plant was removed. Harvesting began from the ripening of the first fruit cluster, with harvests every three days. The fresh weight of the fruit picked from each marked plant was recorded at each harvest until all tomatoes were harvested. When harvest was complete, the total weight per plant was calculated and expressed in kg plant^−1^.

#### 2.3.2. Determination of Mineral Elements in Tomato Plants and Fruits

On days 30, 60, 90, and 120 after transplanting, five tomato plants were randomly selected for each treatment (three replicates per treatment). The roots, stems, leaves, and fruits collected at different days were heated at 105 °C and then dried at 80 °C until a constant weight was reached. The samples were ground separately, passed through a 0.25 mm sieve, and stored in self-sealing bags for the determination of mineral element contents. Total N was determined by the Kjeldahl method [25], total P by the molybdenum-antimony colorimetric method [26], and total K, Ca, and Mg were measured by atomic absorption spectrometry using a ZEEnit 700P (Analytik Jena AG, Jena, Germany) [27].

#### 2.3.3. Determination of Substrate Chemical Properties

After the harvest of all fruits, the substrate attached to the root surface was collected for the six treatments (five tomato plants were selected for each treatment, with three replicates). The collected substrate was air-dried, and foreign materials (such as plant root residues) were removed. The substrate was then passed through a 2 mm sieve, thoroughly mixed, and stored in sealed polyethylene bags prior to chemical and mineral analyses. Substrate pH was determined at a 1:5 (substrate:water) suspension using a Shanghai Jingke Leici PHS-3E pH meter (Shanghai, China) according to the protocols described in Soil Agrochemical Analysis [28]. The matrix conductivity was directly determined by referring to the saturation extraction—conductivity method using the Shanghai Jingke DDSJ-308A conductivity meter (Shanghai, China) [29]. A 0.5 g sample of air-dried substrate, sieved to 0.25 mm, was placed in a 100 mL digestion tube, pre-soaked overnight with 5 mL of concentrated HNO_3_, and 2 mL of HClO_4_ were added the next day. The tube was then heated in a graphite block digester (ED36, LabTech, Sorisole, Italy) using the following temperature ramp: 120 °C for 1 h, 160 °C for 2 h, and 190 °C until white fumes ceased. After cooling, the digest was diluted to 50 mL with deionized water and used for subsequent analyses. Substrate N and P were determined by the Kjeldahl method [25] and the molybdenum-antimony colorimetric method [26], respectively. Substrate K, Ca, and Mg were measured by atomic absorption spectrometry using a ZEEnit 700P (Analytik Jena AG, Jena, Germany) atomic absorption spectrometer [27].

#### 2.3.4. Calculation of Nitrogen Fertilizer Use Efficiency

Nitrogen-use parameters were calculated according to the formulas described by Cassman et al. [30] and Achim Dobermann [31].

A = (A1 − A2)/A3

B = (B1 − B2)/A3

C = B1/A3

Notes: A: nitrogen fertilizer uptake rate

A1: Total nitrogen accumulation in the above-ground parts with nitrogen application

A2: Total nitrogen accumulation in the above-ground parts without nitrogen application

A3: Amount of applied nitrogen

B: Agronomic efficiency of applied N

B1: Tomato yield with nitrogen application

B2: Tomato yield without nitrogen application

C: Partial factor productivity from applied N

#### 2.3.5. Determination of Substrate Enzyme Activities

Urease kit (indophenol blue method, GRS-UE-1-M, lot 20230812): phenol ≥ 99% (AR, Sinopharm, Beijing, China), sodium hypochlorite solution 5% (CP, Xilong Science, Shantou, China), Tris-HCl buffer pH 9.0 (≥99%, Amresco, Solon, OH, USA). Sucrase kit (DNS method, GRS-SC-1-M, lot 20230815): 3,5-dinitrosalicylic acid ≥ 98% (AR, Aladdin, Wenatchee, WA, USA), NaOH pellets ≥ 97% (GR, Sinopharm), sucrose ≥ 99.5% (HPLC, Sigma, St. Louis, MO, USA). Nitrate-reductase kit (sulfanilic acid–naphthylamine method, GRS-NR-1-M, lot 20230810): sulfanilic acid ≥ 99% (AR, Macklin, London, UK), naphthylamine ≥ 99% (AR, Aladdin), KNO_3_ ≥ 99% (GR, Sinopharm). Nitrite-reductase kit (Griess method, GRS-NiR-1-M, lot 20230811): sulfanilic acid ≥ 99% (AR, Titan, Bengaluru, India), α-naphthylamine ≥ 98% (AR, Aladdin), NaNO_2_ ≥ 99% (GR, Sinopharm). Catalase kit (high-sensitivity probe method, GRS-CAT-1-M, lot 20230809): chromogenic probe 2,4-dichlorophenol ≥ 98% (HPLC, TCI, Tokyo, Japan), H_2_O_2_ solution 30% (CP, Xilong Science). The specific determination methods are as follows: The activity of substrate urease was measured using the indophenol blue colorimetric method. The NH3-N produced by hydrolysis reacts with hypochlorite and phenol in a strongly alkaline medium to form the water-soluble dye indophenol blue, which has a characteristic light absorption at 578 nm. The activity of substrate sucrase was measured using the 3,5-dinitrosalicylic acid colorimetric method. The reducing sugars produced by the catalytic degradation of sucrose react with 3,5-dinitrosalicylic acid to form a colored amino compound, which has a characteristic light absorption at 540 nm. The activity of substrate nitrate reductase was measured using the sulfanilic acid-naphthylamine colorimetric method. After the catalytic reduction of nitrate (NO_3_^−^) to nitrite (NO_2_^−^), the product reacts with sulfanilic acid and naphthylamine in an acidic environment to form a red azo compound, which has a characteristic light absorption at 540 nm. The activity of substrate nitrite reductase was measured using the Griess reagent colorimetric method. During the catalytic reduction of nitrite (NO_2_^−^) to ammonium (NH_4_^+^) or nitric oxide (NO), the unreacted nitrite reacts with sulfanilic acid and α-naphthylamine to form a pink azo dye, which has a maximum absorption peak at 520 nm. Substrate catalase catalyzes the decomposition of hydrogen peroxide into water and oxygen. The remaining hydrogen peroxide reacts with a highly sensitive chromogenic probe to form a colored substance, which has a maximum absorption peak at 510 nm.

#### 2.3.6. Analysis of Substrate Microbial Diversity

The substrate was sieved, mixed, and stored in bags with three replicates per treatment, and then kept in a −80 °C freezer for microbial sequencing. The microbial sequencing service was commissioned to Majorbio Bio-Pharm Technology Co., Ltd., Shanghai, China. The specific steps are as follows: (1) DNA extraction and PCR products: Total DNA was extracted according to the instructions of the E.Z.N.A.^®^ Substrate DNA Kit (Omega Bio-tek, Norcross, GA, USA). The concentration and purity of DNA were detected using a NanoDrop 2000 spectrophotometer (Thermo Fisher Scientific, Wilmington, DE, USA), and the quality of DNA extraction was checked by 1% agarose gel electrophoresis. Taking the DNA extracted above as the template, The V3–V4 variable region of the 16S rRNA gene was amplified by PCR using the upstream primers s338F (5′-ACTCCTACGGGAGGCAGCAG-3′) and 806R (5′-GGACTACHVGGGTW TCTAAT-3′)carrying Barcode sequences. The ITS2 region of fungi was amplified using primers ITS1F (5′-CTTGGTCATTTAGAGGAAGTAA 3′) and ITS2R (5′-GCTGCGTTCTTCATCGATGC-3′). These primers have been widely used in high-throughput sequencing research and are regarded as standard primer pairs for analyzing bacterial and fungal communities in matrix and rhizosphere samples. (2) Illumina Miseq sequencing: The PCR products were recovered using 2% agarose gel and purified with the AxyPrep DNA Gel Extraction Kit (Axygen Biosciences, Union City, CA, USA), eluted with Tris-HCl, and detected by 2% agarose gel electrophoresis. Quantitative detection was performed using QuantiFluor™-ST (Promega, Madison, WI, USA). According to the standard operating procedure of the Illumina MiSeq platform (Illumina, San Diego, CA, USA), a PE2*300 library was constructed from the purified amplicons, and then sequencing was carried out on the Illumina Miseq PE300 platform. USEARCH (version 10.0) was used to cluster sequences into OTUs at a similarity level of 97%, and QIIME2 (version 2024.6) was used for species annotation, abundance analysis, and alpha diversity calculation to obtain information on species richness and evenness within samples. Multiple sequence alignments were performed on OTUs, and the differences in community structure among different treatments were analyzed using bar charts.

### 2.4. Statistical Analysis

All experimental data were analyzed using Excel 2021 and SPSS 26.0, and figures were generated with Origin 2021. Prior to analysis of variance, the normality of residuals was assessed using the Shapiro–Wilk test, and the homogeneity of variances was evaluated using Levene’s test. The results indicated that the data satisfied the assumptions of normality and homoscedasticity. Subsequently, one-way analysis of variance (ANOVA) was performed, followed by Duncan’s multiple range test to determine significant differences among treatments at *p* < 0.05. Substrate microbial data were analyzed for species annotation and assessment, species composition analysis, comparative analysis, differential analysis, and correlation analysis with environmental factors using the “Majorbio Cloud Platform.” Different-treatment-related microbial plots were generated using the platform module.

## 3. Results

### 3.1. Effects of Different Nitrogen Forms on Tomato Yield per Plant

**Figure 1:**
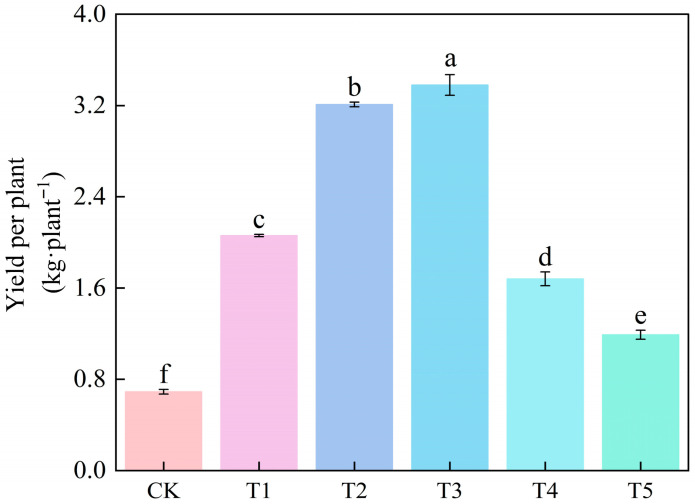
Effects of different treatments on tomato yield per plant. The short vertical lines indicate the mean ± standard error (*n* = 3), and different letters indicate significant differences at the 0.05 level.

The combined nitrogen application in treatment T3 significantly increased the yield per tomato plant compared to other treatments. Under treatment T3, the yield per tomato plant increased by 390.69%, 64.04%, and 5.10% compared to CK, T1, and T2 treatments, respectively. However, compared to T1 and T2, treatments T4 and T5 significantly decreased the yield per tomato plant (Figure 1).

### 3.2. Effects of Different Nitrogen Forms on Tomato Fertilizer Use Efficiency

The nitrogen fertilizer uptake rate, partial factor productivity from applied N, and agronomic efficiency of applied N differed significantly among nitrogen treatments (*p* < 0.05), ranging from 13.10% to 18.07%, 166.90 to 473.49 kg kg^−1^, and 70.41 to 377.00 kg kg^−1^, respectively. Among all treatments, T3 showed significantly higher nitrogen fertilizer uptake rate, partial factor productivity of nitrogen, and agronomic efficiency of nitrogen than the other treatments (*p* < 0.05). Compared to T1, T3 significantly increased the nitrogen fertilizer uptake rate, partial factor productivity of nitrogen, and agronomic efficiency of nitrogen by 17.03%, 64.04%, and 96.20%, respectively (*p* **<** 0.05). Compared to T2, T3 also significantly increased the nitrogen fertilizer uptake rate, partial factor productivity of nitrogen, and agronomic efficiency of nitrogen by 24.87%, 5.10%, and 6.40%, respectively (*p* < 0.05) (Table 3).

### 3.3. Effects of Different Nitrogen Forms on the Enzyme Activities in the Rhizosphere Substrate of Tomato

**Figure 2:**
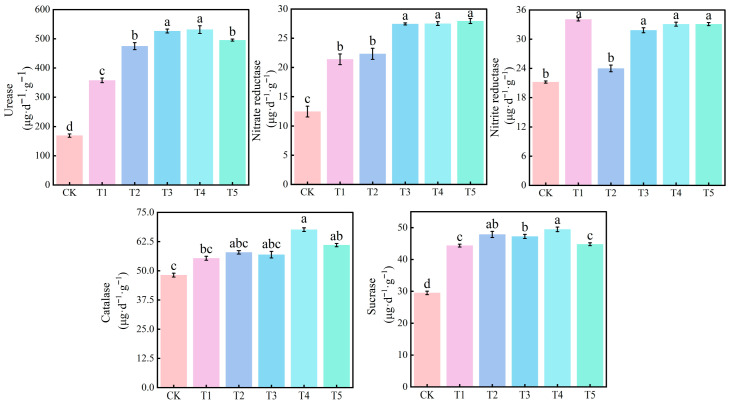
Effects of different treatments on rhizosphere enzyme activities in tomato. urease (URE), nitrate reductase (NR), nitrite reductase (NiR), catalase (CAT), and sucrase (SUC) in the rhizosphere substrate of tomato. The short vertical lines on the bars indicate the mean ± standard error (*n* = 3), and different letters indicate significant differences at the 0.05 level.

The application of nitrogen significantly enhanced the activities of urease, nitrate reductase, nitrite reductase, catalase, and sucrase in the rhizosphere substrate of tomato plants. Among these, the substrate urease activity in treatments T3 and T4 was significantly higher than in other treatments. Specifically, the substrate urease activity in T3 increased by 211.62%, 47.30%, and 10.91% compared to CK, T1, and T2 treatments, respectively. The substrate urease activity in T4 increased by 214.46%, 48.64%, and 11.92% compared to CK, T1, and T2 treatments, respectively. Additionally, the activities of nitrate reductase in the substrate under T3, T4, and T5 treatments were significantly higher than those under CK, T1, and T2 treatments. The activities of nitrite reductase under T3, T4, and T5 treatments were significantly higher than those under CK and T2 treatments, but no significant difference was observed compared to T1 treatment. The activities of catalase and sucrase in the substrate under T4 treatment were the highest, which were significantly higher than those under CK and T1 treatments, but no significant difference was observed compared to T2 treatment. The substrate catalase activity under T4 treatment was significantly increased by 40.42% and 22.15% compared to CK and T1 treatments, respectively. The substrate sucrase activity under T4 treatment was significantly increased by 67.63% and 11.49% compared to CK and T1 treatments, respectively (Figure 2).

### 3.4. Effects of Combined Application of Different Nitrogen Forms on the Nutrient Content of the Rhizosphere Substrate of Tomato

The combined nitrogen application treatments (T3, T4, T5) had significantly higher substrate total nitrogen and available nitrogen contents compared to CK, T1, and T2 treatments. However, the substrate total phosphorus and available phosphorus contents under T3, T4, and T5 treatments were significantly lower than those under CK, T1, and T2 treatments. Compared to CK, the nitrogen application treatments increased the substrate total potassium content but decreased the substrate available potassium, calcium, and magnesium contents as well as the substrate pH value. Additionally, the nitrogen application treatments increased the substrate electrical conductivity (EC), with the highest EC observed under T3 treatment, which increased by 44.84%, 27.04%, and 44.84% compared to CK, T1, and T2 treatments, respectively (Table 4).

### 3.5. Effects of Different Nitrogen Forms on the Accumulation and Distribution of Nitrogen, Phosphorus, Potassium, Calcium, and Magnesium in Plants

#### 3.5.1. Nitrogen Uptake and Allocation

**Figure 3:**
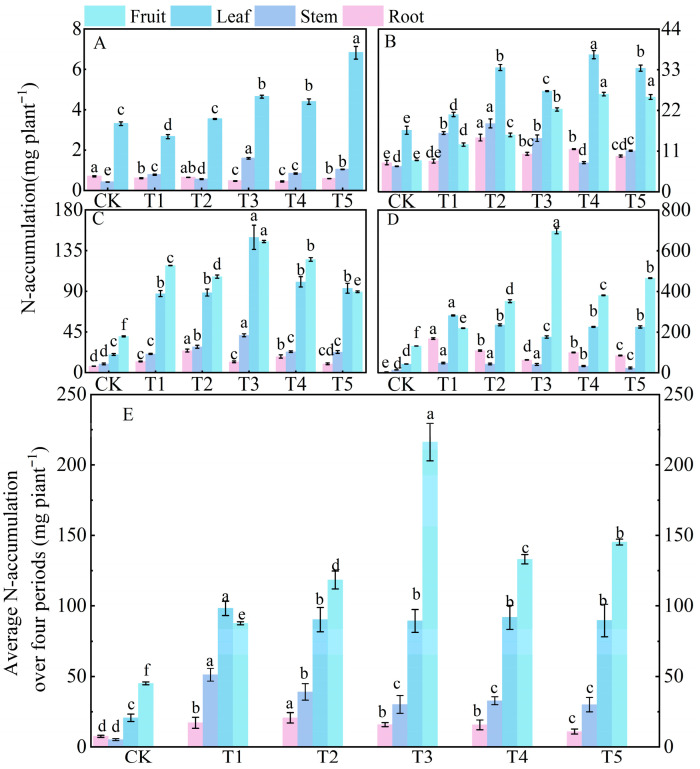
Effects of different treatments on nitrogen accumulation in roots, stems, leaves, and fruits of tomato at different growth days after sowing. Figure (**A**–**D**) show the data of nitrogen accumulation at 30, 60, 90, and 120 days under different treatments, respectively. Figure (**E**) shows the average nitrogen accumulation over 30, 60, 90, and 120 days under different treatments. The short vertical lines on the bars represent the mean ± standard error (*n* = 3), and different letters indicate that the differences between different organs are significant at the 0.05 level.

Compared to the control (CK) treatment, nitrogen application significantly increased nitrogen accumulation in tomato plants throughout their entire growth period. At 30 days after sowing (vegetative growth stage), nitrogen was mainly accumulated in the leaves, with the order of nitrogen accumulation in organs being leaf > stem > root (Figure 3A). The nitrogen accumulation in leaves under treatments T3, T4, and T5 was significantly higher than that under CK, T1, and T2 treatments. At 60 days (transition from vegetative to reproductive growth), the total nitrogen accumulation in plants increased 12 times compared to that at 30 days, with nitrogen beginning to be translocated to the fruits. The order of nitrogen accumulation in organs was leaf > fruit > stem > root (Figure 3B). At 90 days (reproductive growth stage), the nitrogen allocation pattern changed significantly: the order of nitrogen accumulation in organs was fruit > leaf > stem > root (Figure 3C), with the combined nitrogen accumulation in fruits and leaves accounting for 83.56% of the total nitrogen accumulation in plants at 90 days. At 120 days (late reproductive growth stage), treatment T3 had the highest total nitrogen accumulation, which increased by 405.43%, 36.42%, and 32.34% compared to CK, T1, and T2 treatments, respectively (Figure 3D). At this stage, nitrogen accumulated mainly in the fruits, accounting for 53.95% of the total nitrogen accumulation in plants at 120 days. Over the entire growth period, the order of nitrogen accumulation was fruit > leaf > stem > root.

#### 3.5.2. Potassium Uptake and Allocation

**Figure 4:**
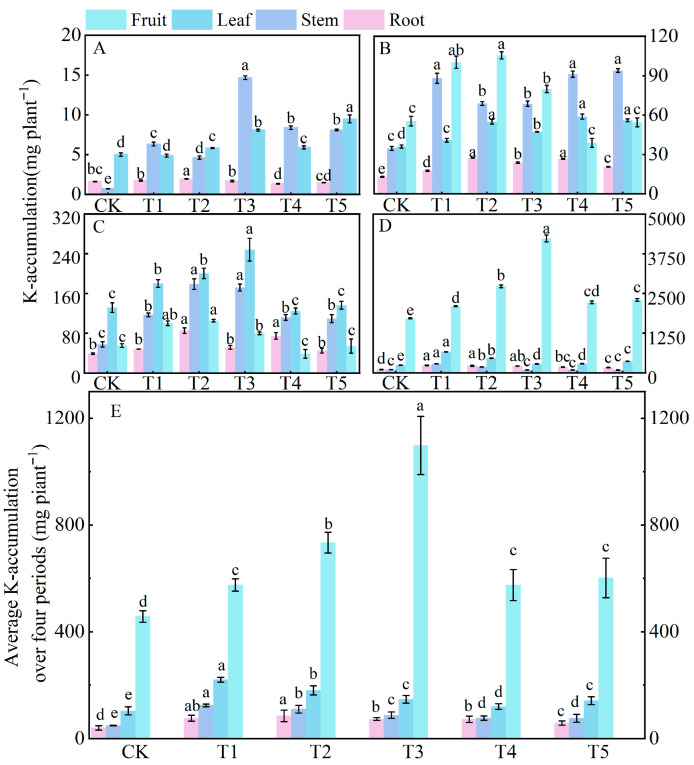
Effects of different treatments on potassium accumulation in roots, stems, leaves, and fruits of tomato at different growth days after sowing. Figure (**A**–**D**) show the data of potassium accumulation at 30, 60, 90, and 120 days under different treatments, respectively. Figure (**E**) shows the average potassium accumulation over 30, 60, 90, and 120 days under different treatments. The short vertical lines on the bars represent the mean ± standard error (*n* = 3), and different letters indicate that the differences between different organs are significant at the 0.05 level.

Compared to the control (CK) treatment, nitrogen application significantly increased potassium accumulation in tomato plants throughout their entire growth period. At 30 days after sowing (the early stage of vegetative growth), potassium was mainly accumulated in the stems, with the order of potassium accumulation in different organs being stem > leaf > root (Figure 4A). The potassium accumulation in the stems under treatments T3, T4, and T5 was significantly higher than that under CK, T1, and T2 treatments. At 60 days (the transition from vegetative to reproductive growth), the total potassium accumulation in plants increased 14-fold compared to that at 30 days. At this time, potassium began to be transported to the fruits, and the order of potassium accumulation in different organs was stem > fruit > leaf > root (Figure 4B). At 90 days (the middle stage of reproductive growth), there was a significant change in the allocation of potassium: the order of potassium accumulation in different organs became fruit > leaf > stem > root (Figure 4C), with the combined potassium accumulation in fruits and leaves accounting for 82.26% of the total potassium accumulation in plants at 90 days. At 120 days (the late stage of reproductive growth), the total potassium accumulation under treatment T3 reached the highest value, which increased by 122.90%, 47.42%, and 34.19% compared to CK, T1, and T2 treatments, respectively (Figure 4D). During this stage, potassium accumulated mainly in the fruits, accounting for 78.17% of the total potassium accumulation. Over the entire growth period, the order of potassium accumulation was fruit > leaf > stem > root.

#### 3.5.3. Phosphorus Uptake and Allocation

**Figure 5:**
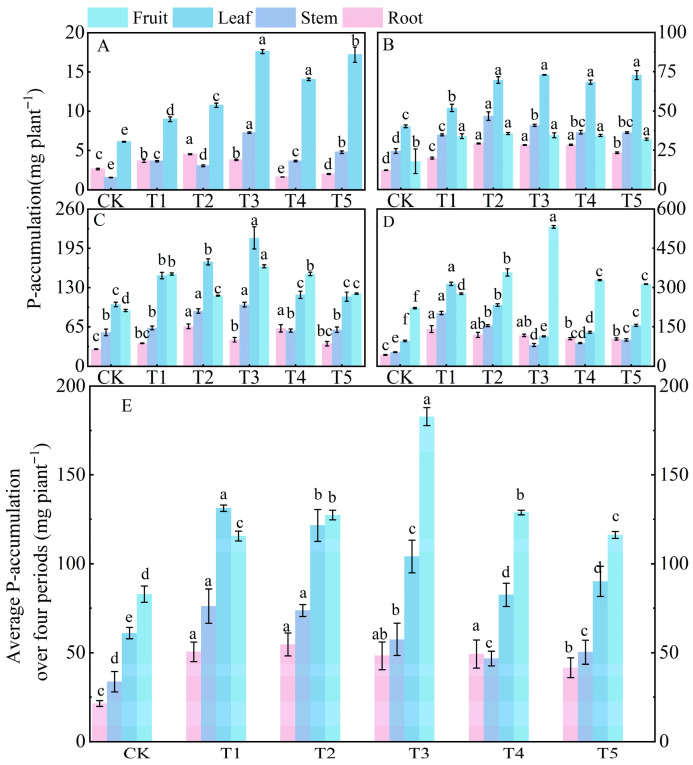
Effects of different treatments on phosphorus accumulation in roots, stems, leaves, and fruits of tomato at different growth days after sowing. Figure (**A**–**D**) show the data of phosphorus accumulation at 30, 60, 90, and 120 days under different treatments, respectively. Figure (**E**) shows the average phosphorus accumulation over 30, 60, 90, and 120 days under different treatments. The short vertical lines on the bars represent the mean ± standard error (*n* = 3), and different letters indicate that the differences between different organs are significant at the 0.05 level.

Compared to the control (CK) treatment, nitrogen application significantly increased phosphorus accumulation in tomato plants throughout their entire growth period. Compared to nitrogen and potassium, the total accumulation of phosphorus was relatively lower. At 30 days after sowing (the early stage of vegetative growth), phosphorus was mainly accumulated in the leaves, with the order of phosphorus accumulation in different organs being leaf > stem > root (Figure 5A). The phosphorus accumulation in the leaves under treatments T3, T4, and T5 was significantly higher than that under CK, T1, and T2 treatments. At 60 days (the transition from vegetative to reproductive growth), the total phosphorus accumulation in plants increased 7.9 times compared to that at 30 days. At this time, phosphorus began to be transported to the fruits, and the order of phosphorus accumulation in different organs was leaf > fruit > stem > root (Figure 5B). At 90 days (the middle stage of reproductive growth), there was a shift in the allocation pattern of phosphorus, with the order of phosphorus accumulation becoming fruit > leaf > stem > root (Figure 5C). The combined phosphorus accumulation in leaves and fruits accounted for 70.26% of the total phosphorus accumulation in plants at 90 days. At 120 days (the late stage of reproductive growth), the total phosphorus accumulation under treatment T3 increased by 104.10%, 9.76%, and 2.42% compared to CK, T1, and T2 treatments, respectively (Figure 5D). During this stage, phosphorus accumulated mainly in the fruits, accounting for 46.34% of the total phosphorus accumulation. Overall, the order of phosphorus accumulation over the entire growth period was fruit > leaf > stem > root.

#### 3.5.4. Calcium and Magnesium Uptake and Allocation

**Figure 6:**
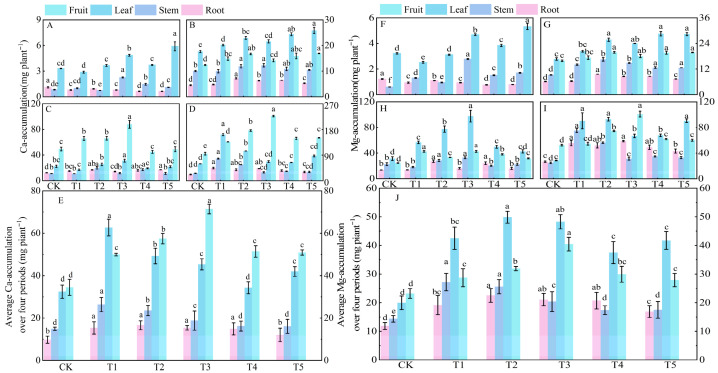
Effects of different treatments on calcium and magnesium accumulation in roots, stems, leaves, and fruits of tomato at different growth days after sowing. Figure (**A**–**D**) show the data of calcium accumulation at 30, 60, 90, and 120 days under different treatments, respectively. Figure (**E**) shows the average calcium accumulation over 30, 60, 90, and 120 days under different treatments. Figure (**F**–**I**) show the data of magnesium accumulation at 30, 60, 90, and 120 days under different treatments, respectively. Figure (**J**) shows the average magnesium accumulation over 30, 60, 90, and 120 days under different treatments. The short vertical lines on the bars represent the mean ± standard error (=3), and different letters indicate that the differences between different organs are significant at the 0.05 level.

Compared to the control (CK) treatment, nitrogen application significantly increased calcium accumulation in tomato plants throughout their entire growth period. At 30 days after sowing (the early stage of vegetative growth), calcium was mainly accumulated in the leaves, with the order of calcium accumulation in different organs being leaf > stem > root (Figure 6A). The calcium accumulation in the leaves under treatment T5 reached the highest value, followed by treatment T3. At 60 days (the transition from vegetative to reproductive growth), the total calcium accumulation in plants increased 7.5 times compared to that at 30 days. At this time, calcium began to be transported to the fruits, and the order of calcium accumulation in different organs was leaf > fruit > stem > root (Figure 6B). At 90 days, the calcium accumulation in fruits increased rapidly, while the calcium accumulation in leaves, stems, and roots decreased, with the order of calcium accumulation in different organs being fruit > leaf > stem > root (Figure 6C). At 120 days (the late stage of reproductive growth), the total calcium accumulation under treatment T1 reached the highest, which increased by 107.00% compared to CK (Figure 6D). Over the entire growth period, the order of calcium accumulation was fruit > leaf > stem > root.

Compared to the CK treatment, the combined application of different magnesium forms significantly increased the magnesium accumulation in tomato plants throughout their entire growth period. At 30 days after sowing (the early stage of vegetative growth), magnesium was mainly accumulated in the leaves, with the order of magnesium accumulation in different organs being leaf > stem > root (Figure 6E). The magnesium accumulation in the leaves under treatments T3, T4, and T5 was significantly higher than that under CK, T1, and T2 treatments. At 60 days, the total magnesium accumulation in plants increased 8.9 times compared to that at 30 days. During this period, magnesium began to be allocated to the fruits, and the order of magnesium accumulation in different organs was leaf > fruit > stem > root (Figure 6F). At 90 days, the magnesium accumulation in leaves increased rapidly, especially under treatment T3, with the order of magnesium accumulation in different organs being leaf > fruit > stem > root (Figure 6G). At 120 days, the total magnesium accumulation under treatment T3 reached the highest, which increased by 83.24%, 2.70%, and 4.32% compared to CK, T1, and T2 treatments, respectively (Figure 6H). During this stage, magnesium accumulated mainly in leaves and fruits, accounting for 60.86% of the total magnesium accumulation. Over the entire growth period, the order of magnesium accumulation was leaf > fruit > stem > root.

### 3.6. Effects of Different Nitrogen Forms on the Rhizosphere Microbial Community of Tomato

#### 3.6.1. Analysis of Differences in Rhizosphere Microbial Communities Under Different Nitrogen Treatments

**Figure 7:**
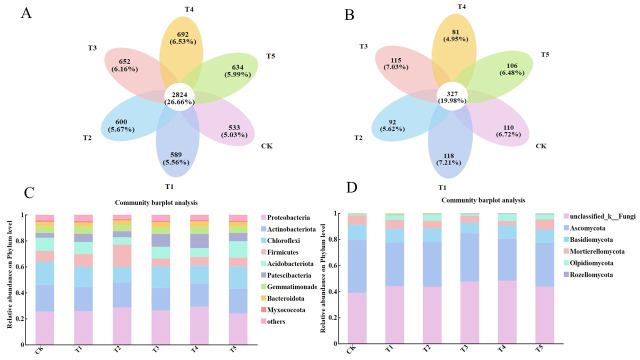
Venn diagrams of bacterial (**A**) and fungal (**B**) communities in the rhizosphere substrate under different treatments, and the phylum-level composition of bacterial (**C**) and fungal (**D**) communities in the rhizosphere substrate under different treatments.

The results of Venn diagram analysis showed that the combined application of ammonium nitrogen and urea-N significantly changed the operational taxonomic unit (OTU) composition characteristics of substrate microbial communities. In the bacterial community (Figure 7A), the number of specific bacteria was the highest under treatment T4, followed by treatment T3. Compared to CK, the number of specific bacteria increased by 29.83% and 22.33%, respectively. In the fungal community (Figure 7B), the number of specific fungi was the highest under treatment T1, followed by treatment T3. Compared to CK, the number of specific fungi increased by 7.27% and 4.55%, respectively. It is worth noting that treatment T3 showed the second-highest number of specific microorganisms in both bacterial and fungal communities.

Based on the relative abundance of the top 10 species at the phylum level, the community structure distribution of the rhizosphere substrate under different treatments was plotted. It was found that the community composition under different treatments showed significant similarity, but there were differences in the proportion of dominant bacterial groups (Figure 7C,D). Analysis of the bacterial community showed that Proteobacteria, Actinobacteriota, Chloroflexi, Firmicutes, and Acidobacteriota were the top 5 bacterial groups in terms of abundance under each treatment (relative abundance ranging from 5.5% to 12.6%), accounting for more than 75% of the total bacterial population (Figure 7C). Analysis of the fungal community showed that unclassified_k__Fungi, Ascomycota, Basidiomycota, Mortierellomycota, and Olpidiomycota were the top 5 fungal groups in terms of abundance under each treatment (relative abundance > 5%), accounting for more than 75% of the total fungal population (Figure 7D).

#### 3.6.2. Beta Diversity of Rhizosphere Microbial Communities Under Different Nitrogen Treatments

**Figure 8:**
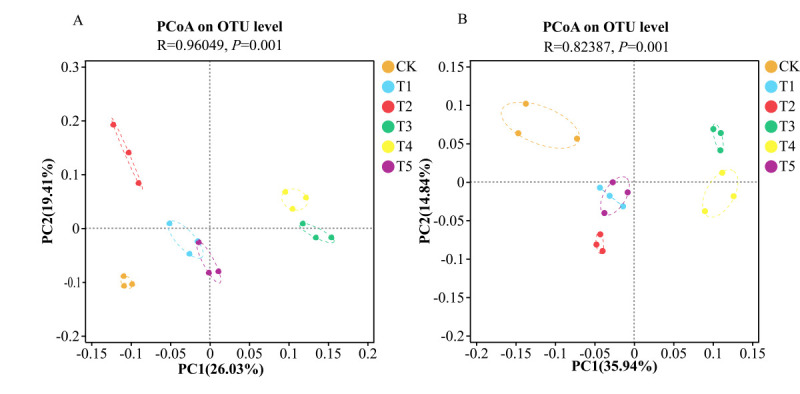
PCoA analysis of bacterial (**A**) and fungal (**B**) communities in the substrate under different treatments. The dots within each circle represent the individual replicate values for that treatment, showing the spread and distribution of the data.

The combined application of different nitrogen forms significantly affected the composition of bacterial and fungal communities in the rhizosphere at the OTU (Operational Taxonomic Units) level. For bacterial communities (Figure 8A), the contributions of the first and second principal components were 26.03% and 19.41%, respectively, with a cumulative contribution of 45.44%. For fungal communities (Figure 8B), the contributions of the first and second principal components were 35.94% and 14.84%, respectively, with a cumulative contribution of 50.78%. PCoA (Principal Coordinates Analysis) showed that the three replicates of each treatment were close to each other, indicating good reproducibility. For both bacterial and fungal communities, PC1 could clearly separate the treatments T3 and T4 from the other treatments. The microbial communities of these two groups were located in different quadrants, indicating significant differences in community structure.

#### 3.6.3. Correlation Analysis Between Substrate Microbial Communities and Substrate Environmental Factors Under Different Nitrogen Treatments

**Figure 9:**
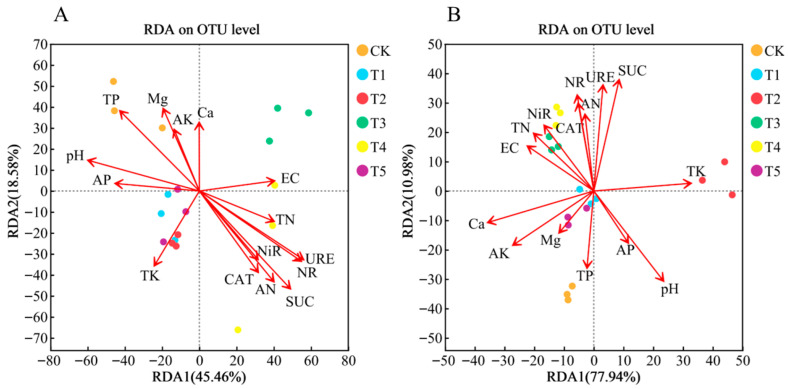
Redundancy analysis (RDA) of substrate bacteria (**A**) and fungi (**B**) under different treatments. Notes: urease (URE), nitrate reductase (NR), nitrite reductase (NiR), catalase (CAT), and sucrase (SUC), total nitrogen (TN), total phosphorus (TP), total potassium (TK), calcium (Ca), magnesium (Mg), available nitrogen (AN), available phosphorus (AP), available potassium (AK), electrical conductivity (EC), and substrate pH. The angle between the environmental factor arrow and the sample point (acute angle indicates positive correlation, obtuse angle indicates negative correlation), the longer the arrow, the stronger the correlation.

As shown in Figure 9, at the OTU level of substrate bacterial and fungal communities, the explanatory power of substrate environmental factors was 64.04% and 88.92%, respectively. Ca, Mg, TP, AP, AK, TK, and pH were positively correlated with the substrate bacterial and fungal communities under treatments CK, T1, T2, and T5, and negatively correlated with the substrate bacterial and fungal communities under treatments T3 and T4. In contrast, SUC, CAT, NiR, NR, URE, TN, and EC were positively correlated with the substrate bacterial and fungal communities under treatments T3 and T4, and negatively correlated with those under treatments CK, T1, T2, and T5.

#### 3.6.4. Lefse Analysis of Differences in Microbial Taxa Under Different Nitrogen Treatments

**Figure 10:**
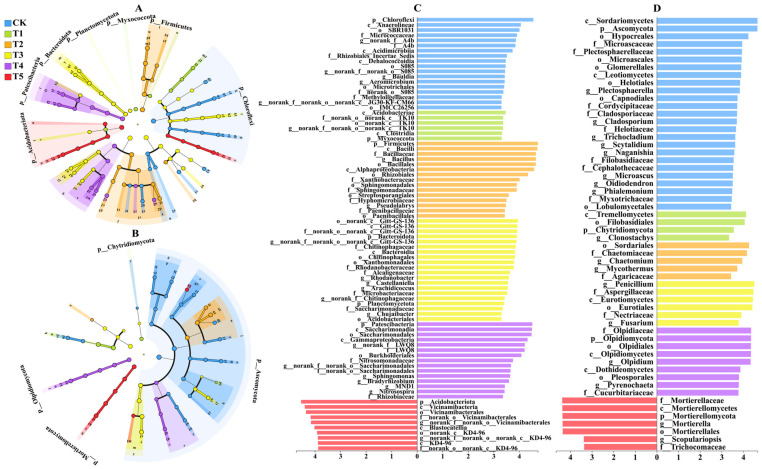
LefSe diagrams of bacterial (**A**) and fungal (**B**) communities under different treatments, and LDA discrimination results of bacterial (**C**) and fungal (**D**) communities. The LDA bar chart represents the matrix microbial groups that show significant differences under different treatments.

LEfSe (Linear Discriminant Analysis Effect Size) is a statistical method used to identify microbial taxa that exhibit significant differences between multiple groups and have sufficiently large effect sizes. It first uses a non-parametric Kruskal–Wallis test to detect significant differences between groups, and then applies LDA (Linear Discriminant Analysis) to estimate the magnitude (effect size) of these differences. In this way, LEfSe screens for biomarkers that are significantly enriched in abundance under specific treatments/environments and contribute most to the differentiation between groups. This study used LEfSe analysis based on LDA (threshold 3.3) to compare groups in the rhizosphere substrate under different nitrogen treatments and identify differentially abundant microbial taxa. The results showed that the number of significantly different bacteria and fungi in the substrate under the T3 treatment increased significantly compared to single nitrogen source treatments. Regarding bacteria, the substrate samples from the CK, T1, T2, T3, T4, and T5 treatments were enriched with 18, 6, 15, 20, 15, and 10 significantly different bacterial communities, respectively (Figure 10A,C). Regarding fungi, the substrate samples treated with CK, T1, T2, T3, T4, and T5 were enriched with 25, 4, 5, 6, 9, and 7 significantly different fungal communities, respectively (Figure 10B,D).

## 4. Discussion

The results showed that the combined application of ammonium nitrogen (AN) and amide nitrogen (UN) significantly improved tomato yield, nitrogen fertilizer utilization and substrate properties, with the T3 treatment (25% AN + 75% UN) showing the best overall performance. This indicates that a suitable AN:UN ratio can better meet the nitrogen requirements of tomatoes at different growth stages. In terms of growth and nitrogen fertilizer absorption, the T3 treatment significantly improved the yield per plant and nitrogen fertilizer absorption in the later harvest period (120 days). This may be due to the synergistic nitrogen supply mechanism of AN and UN: AN can be directly absorbed, which is beneficial to the rapid growth of seedlings [32], while UN is slowly hydrolyzed by urease, continuously releasing NH_4_^+^ [33], providing a stable nitrogen source for the middle and late growth stages and avoiding temporary nitrogen deficiency or loss. Anderson J [34] reported that an appropriate amount of UN can improve crop dry matter accumulation and nitrogen fertilizer utilization, which is consistent with our T3 treatment results. Regarding substrate nutrients, the total nitrogen and alkaline hydrolyzable nitrogen contents of treatments T3, T4, and T5 were all increased compared to CK, T1, and T2. This is related to the conversion characteristics of different nitrogen sources: AN is easily adsorbed by colloids and is not easily leached [35], while UN hydrolysis continuously replenishes NH_4_^+^, improving the nitrogen supply capacity of the soil [36]. Some of the NH_4_^+^ released from UN will be nitrified, releasing H^+^, thereby reducing the substrate pH [37,38]. Compared to CK, the decrease in total phosphorus, available phosphorus, available potassium, calcium, and magnesium contents under nitrogen application treatment may be due to the plants absorbing a large amount of substrate nutrients for their growth and development, or it may be due to the acidification of the rhizosphere substrate. Soil acidification enhances the activity of Fe^3+^ and Al^3+^, forming insoluble phosphates (AlPO_4_, FePO_4_), and reduces the availability of phosphorus [39,40]. NH_4_^+^ and K^+^ have similar charges and ionic radii, and they compete for adsorption on the surface of the soil colloidal layer, resulting in a low content of K^+^ in the soil [41]. There is an antagonistic effect between NH_4_^+^, Ca^2+^, and Mg^2+^, causing NH_4_^+^ to occupy the adsorption sites of divalent cations through electrostatic interactions, leading to the release of Ca^2+^ and Mg^2+^ in the solution and their migration with water [40]. Regarding enzyme activity, the application of amide nitrogen significantly increased the activities of urease, nitrate reductase, nitrite reductase, catalase, and sucrase, especially under T3 treatment. The hydrolysis of amide nitrogen depends on the action of urease [42], and the activities of urease, nitrate reductase, and nitrite reductase in soil treated with amide nitrogen are enhanced [43]. Tong [44] also found that different nitrogen combinations significantly increased the activities of catalase and sucrase in the soil, consistent with our results. In terms of mineral element absorption, compared to single nitrogen fertilizer treatment, T3 treatment significantly increased the accumulation of N, P, K, Ca and Mg in tomato plants, and these elements were gradually transported to the fruit in the later stages of growth. The synergistic supply of two nitrogen forms in T3 treatment regulated the carbon-nitrogen metabolic balance and may have enhanced the activity of nitrogen assimilation enzymes such as glutamine synthase/glutamate synthase (GS/GOGAT) [45]. At the same time, the slow release of amide nitrogen avoided proton toxicity caused by excessive NH_4_^+^ and provided more ATP and carbon skeleton for mineral element absorption [45]. Fang [46] also reported that sufficient nitrogen fertilizer supply can activate the expression of AMT2 gene and synergistically improve the absorption efficiency of mineral elements, which is consistent with our results. Regarding the microbial community structure, principal coordinate analysis (PCoA) showed obvious separation between different nitrogen fertilizer treatments, with T3 and T4 forming an independent cluster. RDA analysis further showed that the microbial community under T3 and T4 treatments was positively correlated with total nitrogen content, conductivity and the activity of various enzymes. The studies by Yin [47] and Fierer [48] showed that nitrogen fertilizer application can alter the physicochemical properties and enzyme activity of soil, thereby driving changes in the microbial community structure. The LDA discriminant results showed that the T3 treatment enriched bacterial groups with carbon and nitrogen cycling functions as well as potential growth-promoting fungi (g_Penicillium). Hassine [49]’s research also indicated that these microorganisms may promote tomato growth and yield formation by improving nutrient conversion efficiency and the rhizosphere environment, which is consistent with the results of this experiment.

Therefore, our results demonstrate that balanced co-application of AN and UN, especially the T3 ratio, simultaneously improves substrate nutrient status, enzyme activities and microbial community structure, leading to higher tomato yield and N-use efficiency. These results are consistent with previous studies and may provide a reliable basis for optimizing the form and proportion of nitrogen in greenhouse tomato substrate cultivation.

## 5. Conclusions

This study used tomatoes as the experimental material to systematically compare the comprehensive effects of different ratios of ammonium nitrogen (AN) and amide nitrogen (UN) mixtures on tomato yield, mineral element accumulation, substrate microbial community, and substrate enzyme activity. Compared to single nitrogen sources, all AN-UN mixtures increased per-plant yield, mineral element uptake (N, P, K, Mg), and nitrogen fertilizer absorption rate while also promoting substrate microbial abundance and substrate enzyme activity. The 25% AN + 75% UN treatment (T3) showed the most significant effect. This study utilized the characteristic that amide nitrogen can slowly decompose into nitrate nitrogen, and investigated the effects of mixed application of ammonium and amide nitrogen. It was found that the mixed application of ammonium and amide nitrogen improved substrate nutrients and altered the substrate microbial environment, thereby increasing tomato yield. This may provide a practical solution for optimizing nitrogen fertilizer forms in greenhouse tomato cultivation. Currently, the results of this study are based only on a single variety and a single pot substrate; this fertilization scheme has not yet been validated in different varieties and long-term field trials. Future research will focus more on the regulatory effect of the T3 treatment on tomato quality and validate these conclusions in different varieties and long-term field trials.

## Figures and Tables

**Table 1:** Physical and chemical properties of the substrate.

Substrate	AN (mg kg^−1^)	AK (g kg^−1^)	AP (g kg^−1^)	TN (g kg^−1^)	TK (g kg^−1^)	TP (g kg^−1^)	EC (mS cm^−1^)	Bulk Density (g cm^−3^)	pH
substrate:peat:vermiculite = 2:1:1	429.77	0.36	0.90	1.96	0.90	0.64	1.56	0.54	6.49

Note: AN: Available N, AK: Available K, AP: Available P, TN: Total N, TK: Total K, TP: Total P. The substrate was purchased from Gansu Lv neng Ruiqi Agricultural Technology Co., Ltd. (Wuwei, China).

**Table 2:** Different forms of nitrogen ratios and concentrations of major elements in nutrient solutions.

Treatments	Nitrogen Form and Ratio	Elements Concentration (mmol L^−1^)					
		NH_4_^+^-N	CO(NH_2_)_2_	P	K	Ca	Mg
CK	-	0	0	1	6	5	2
T1	NH_4_^+^-N (AN)	15	0	1	6	5	2
T2	Urea (UN)	0	15	1	6	5	2
T3	25%AN:75%UN	3.75	11.25	1	6	5	2
T4	50%AN:50%UN	7.5	7.5	1	6	5	2
T5	75%AN:25%UN	11.25	3.75	1	6	5	2

**Table 3:** Effects of different treatments on nitrogen fertilizer use efficiency in tomato.

Treatments	Nitrogen Fertilizer Uptake Rate (%)	Partial Factor Productivity from Applied N (kg kg^−1^)	Agronomic Efficiency of Applied N (kg kg^−1^)
T1	15.44 ± 0.21 b	288.64 ± 1.95 c	192.15 ± 1.95 c
T2	14.47 ± 0.34 c	450.49 ± 2.36 b	354.00 ± 2.36 b
T3	18.07 ± 0.34 a	473.49 ± 11.98 a	377.00 ± 11.98 a
T4	13.10 ± 0.18 d	235.34 ± 8.43 d	138.85 ± 8.43 d
T5	15.74 ± 0.16 b	166.90 ± 6.10 e	70.41 ± 6.10 e

Notes: Data are presented as mean ± standard error (*n* = 5). Different lowercase letters within the same column indicate significant differences among treatments at *p* < 0.05 (one-way ANOVA followed by post hoc test).

**Table 4:** Effects of different treatments on nutrient content in the rhizosphere substrate of tomato.

Treatments	TN (g kg^−1^)	TP (g kg^−1^)	TK (g kg^−1^)	Ca (g kg^−1^)	Mg (g kg^−1^)	AN (mg kg^−1^)	AP (mg kg^−1^)	AK (mg kg^−1^)	EC (mS cm^−1^)	pH
CK	3.78 ± 0.03 d	1.71 ± 0.09 a	10.08 ± 0.20 b	2.01 ± 0.06 a	2.01 ± 0.07 a	278.10 ± 13.45 d	0.64 ± 0.02 ab	3.08 ± 0.07 a	1.07 ± 0.04 c	6.44 ± 0.06 a
T1	5.63 ± 0.22 c	1.55 ± 0.01 b	11.39 ± 0.18 a	1.90 ± 0.01 a	1.92 ± 0.01 a	702.77 ± 14.15 bc	0.64 ± 0.01 a	2.75 ± 0.08 b	1.22 ± 0.04 b	5.78 ± 0.12 b
T2	4.48 ± 0.20 d	1.36 ± 0.03 c	11.92 ± 0.19 a	1.68 ± 0.01 b	1.92 ± 0.004 a	644.43 ± 27.29 c	0.60 ± 0.01 b	2.31 ± 0.09 c	1.07 ± 0.05 c	6.28 ± 0.02 a
T3	7.97 ± 0.39 b	1.39 ± 0.03 c	10.15 ± 0.24 b	1.94 ± 0.02 a	1.97 ± 0.001 a	712.10 ± 24.36 b	0.54 ± 0.01 c	2.72 ± 0.09 b	1.55 ± 0.03 a	5.22 ± 0.10 cd
T4	7.54 ± 0.33 b	1.25 ± 0.05 cd	10.17 ± 0.22 b	1.93 ± 0.01 a	1.94 ± 0.01 a	784.43 ± 17.19 a	0.56 ± 0.02 c	2.74 ± 0.13 b	1.31 ± 0.03 b	5.06 ± 0.03 d
T5	9.52 ± 0.17 a	1.21 ± 0.03 d	10.48 ± 0.27 b	1.91 ± 0.05 a	1.93 ± 0.001 a	819.43 ± 18.78 a	0.53 ± 0.01 c	2.68 ± 0.11 b	1.5 ± 0.03 a	5.42 ± 0.07 c

Notes: AN: Available N, AK: Available K, AP: Available P, TN: Total N, TK: Total K, TP: Total P. Data in the table are presented as the mean ± standard error (*n* = 3). Within each column, different letters indicate significant differences at the 0.05 level.

## Data Availability

The data sets generated during and/or analyzed during the current study are available from the corresponding author on reasonable request. Sequencing data are stored in the NCBI database, access link: Bacteria: https://www.ncbi.nlm.nih.gov/sra/PRJNA1398585 (accessed on 6 Jan 2026) Fungi: https://www.ncbi.nlm.nih.gov/sra/PRJNA1398600 (accessed on 6 Jan 2026).

## Abbreviations

The following abbreviations are used in this manuscript:

AK	available potassium
AP	available phosphatase
AN	available nitrogen
URE	urease
NiR	nitrite reductase
SUC	sucrase
OTU	operational taxonomic unit
RDA	redundancy analysis
LEfSe	linear discriminant analysis effect size
TK	total potassium
TP	total phosphorus
TN	available nitrogen
NR	nitrate reductase
CAT	catalase
EC	electrical conductivity
PCoA	principal coordinate analysis
LDA	linear discriminant analysis
